# Key characteristics impacting survival of COVID-19 extracorporeal membrane oxygenation

**DOI:** 10.1186/s13054-022-04053-6

**Published:** 2022-06-28

**Authors:** Johannes Herrmann, Christopher Lotz, Christian Karagiannidis, Steffen Weber-Carstens, Stefan Kluge, Christian Putensen, Andreas Wehrfritz, Karsten Schmidt, Richard K. Ellerkmann, Daniel Oswald, Gösta Lotz, Viviane Zotzmann, Onnen Moerer, Christian Kühn, Matthias Kochanek, Ralf Muellenbach, Matthias Gaertner, Falk Fichtner, Florian Brettner, Michael Findeisen, Markus Heim, Tobias Lahmer, Felix Rosenow, Nils Haake, Philipp M. Lepper, Peter Rosenberger, Stephan Braune, Mirjam Kohls, Peter Heuschmann, Patrick Meybohm, for the German ECMO COVID Study Group, Martha E. Hübsch, Quirin Notz, Daniel Röder, Peter Kranke, Michaela L. Merten, Dominik Jarczak, Kevin Roedl, Jens-Christian Schewe, Stefan F. Ehrentraut, Stefan Kreyer, Ixchel Castellanos, Thorsten Brenner, Frank Herbstreit, Florian Espeter, Jan Wiefhoff, Björn Ellger, Florian J. Raimann, Michael Sonntagbauer, Tobias Wengenmayer, Dawid Staudacher, Ruslan Natanov, Caroline Rolfes, Christian Reyher, Iuliu-Emilian Torje, Patricia Glaser, Vanessa Rembold, Rainer Haseneder, Jan Sackarnd, Johannes Lepper, Andre Becker, Guy Danziger, Carlos Metz, Valbona Mirakaj, Stefanie Calov, Anna Grau, Kirsten Haas, Katrin Ungethüm, Karl Bihlmaier

**Affiliations:** 1 Department of Anaesthesiology, Intensive Care, Emergency and Pain Medicine, University Hospital Wuerzburg, Oberduerrbacherstr. 6, 97080 Würzburg, Germany; 2 Department of Pneumology and Critical Care Medicine, Cologne-Merheim Hospital, ARDS and ECMO Center, Kliniken Der Stadt Köln, Witten/Herdecke University Hospital, Cologne, Germany; 3 Department of Anesthesiology and Operative Intensive Care Medicine (CCM, CVK), Charité - Universitätsmedizin Berlin, Berlin, Germany; 4 Department of Intensive Care, University Medical Center Hamburg-Eppendorf, Hamburg, Germany; 5 Department of Anesthesiology and Intensive Care Medicine, University Hospital Bonn, Bonn, Germany; 6 Department of Anaesthesiology, University Hospital Erlangen, Friedrich-Alexander University, Erlangen-Nuernberg (FAU), Erlangen, Germany; 7 Department of Anesthesiology and Intensive Care Medicine, University Hospital Essen, University Duisburg-Essen, Essen, Germany; 8 Department of Anesthesiology and Intensive Care Medicine, Klinikum Dortmund, Klinikum University Witten/Herdecke, Dortmund, Germany; 9 Department of Anesthesiology, Intensive Care Medicine and Pain Therapy, Clinic Centre Westfalen, Dortmund, Germany; 10 Department of Anaesthesiology, Intensive Care Medicine and Pain Therapy, University Hospital Frankfurt, Goethe University Frankfurt, Frankfurt, Germany; 11 Department of Cardiology and Angiology I (Heart Center Freiburg - Bad Krozingen), Medical Center - University of Freiburg, Faculty of Medicine, University of Freiburg, Freiburg, Germany; 12 Interdisciplinary Medical Intensive Care (IMIT), Medical Center - University of Freiburg, Faculty of Medicine, University of Freiburg, Freiburg, Germany; 13 Department of Anesthesiology, University Medical Center Göttingen, Robert-Koch-Str. 40, 37085 Göttingen, Germany; 14 Department of Cardiothoracic, Transplantation and Vascular Surgery, Hannover Medical School, Hannover, Germany; 15 Department of Internal Medicine, Division I (Hematology/Oncology), University Hospital of Cologne, Cologne, Germany; 16 Department of Anesthesiology and Critical Care Medicine, ARDS/ECMO-Center, Campus Kassel of the University of Southampton, Kassel, Germany; 17 Department of Anaesthesia, Perioperative Medicine and Interdisciplinary Intensive Care Medicine, ECLS/ECMO-Center, Asklepios Klinik Langen, Langen, Germany; 18 Department of Anesthesiology and Intensive Care Medicine, University of Leipzig Medical Center, Leipzig, Germany; 19 ARDS- und ECMO Zentrum München-Nymphenburg, Barmherzige Brüder Krankenhaus München, München, Germany; 20 Klinik für Pneumologie, Internistische Intensiv- und Beatmungsmedizin, München Klinik Harlaching, Munich, Germany; 21 Department of Anaesthesiology and Intensive Care Medicine, Technical University of Munich, School of Medicine, Munich, Germany; 22 School of Medicine, University Hospital Rechts Der Isar, Department of Internal Medicine II, University of Munich, Ismaninger Str. 22, 81675 Munich, Germany; 23 Department of Cardiology I – Coronary and Peripheral Vascular Disease, Heart Failure, University Hospital Muenster, Muenster, Germany; 24 Department of Intensive Care Medicine, Imland Klinik Rendsburg, Rendsburg, Germany; 25 Department of Internal Medicine V- Pneumology, Allergology and Critical Care Medicine, Saarland University, Homburg, Germany; 26 Department of Anesthesiology and Intensive Care Medicine, University Hospital Tübingen, Eberhard Karls University Tübingen, Tübingen, Germany; 27 Department of Medical Intensive Care and Emergency Medicine, St. Franziskus-Hospital Muenster, Münster, Germany; 28 Institute of Clinical Epidemiology and Biometry, University of Würzburg, Würzburg, Germany; 29 Clinical Trial Center Würzburg, Universitätsklinikum Würzburg, Würzburg, Germany

**Keywords:** COVID-19, Acute respiratory distress syndrome (ARDS), Intensive care, Extracorporeal life support, Case-volume relationship

## Abstract

**Background:**

Severe COVID-19 induced acute respiratory distress syndrome (ARDS) often requires extracorporeal membrane oxygenation (ECMO). Recent German health insurance data revealed low ICU survival rates. Patient characteristics and experience of the ECMO center may determine intensive care unit (ICU) survival. The current study aimed to identify factors affecting ICU survival of COVID-19 ECMO patients.

**Methods:**

673 COVID-19 ARDS ECMO patients treated in 26 centers between January 1st 2020 and March 22nd 2021 were included. Data on clinical characteristics, adjunct therapies, complications, and outcome were documented. Block wise logistic regression analysis was applied to identify variables associated with ICU-survival.

**Results:**

Most patients were between 50 and 70 years of age. PaO_2_/FiO_2_ ratio prior to ECMO was 72 mmHg (IQR: 58–99). ICU survival was 31.4%. Survival was significantly lower during the 2nd wave of the COVID-19 pandemic. A subgroup of 284 (42%) patients fulfilling modified EOLIA criteria had a higher survival (38%) (*p* = 0.0014, OR 0.64 (CI 0.41–0.99)). Survival differed between low, intermediate, and high-volume centers with 20%, 30%, and 38%, respectively (*p* = 0.0024). Treatment in high volume centers resulted in an odds ratio of 0.55 (CI 0.28–1.02) compared to low volume centers. Additional factors associated with survival were younger age, shorter time between intubation and ECMO initiation, BMI > 35 (compared to < 25), absence of renal replacement therapy or major bleeding/thromboembolic events.

**Conclusions:**

Structural and patient-related factors, including age, comorbidities and ECMO case volume, determined the survival of COVID-19 ECMO. These factors combined with a more liberal ECMO indication during the 2nd wave may explain the reasonably overall low survival rate. Careful selection of patients and treatment in high volume ECMO centers was associated with higher odds of ICU survival.

***Trial registration*:**

Registered in the German Clinical Trials Register (study ID: DRKS00022964, retrospectively registered, September 7th 2020, https://www.drks.de/drks_web/navigate.do?navigationId=trial.HTML&TRIAL_ID=DRKS00022964.

**Graphical abstract:**

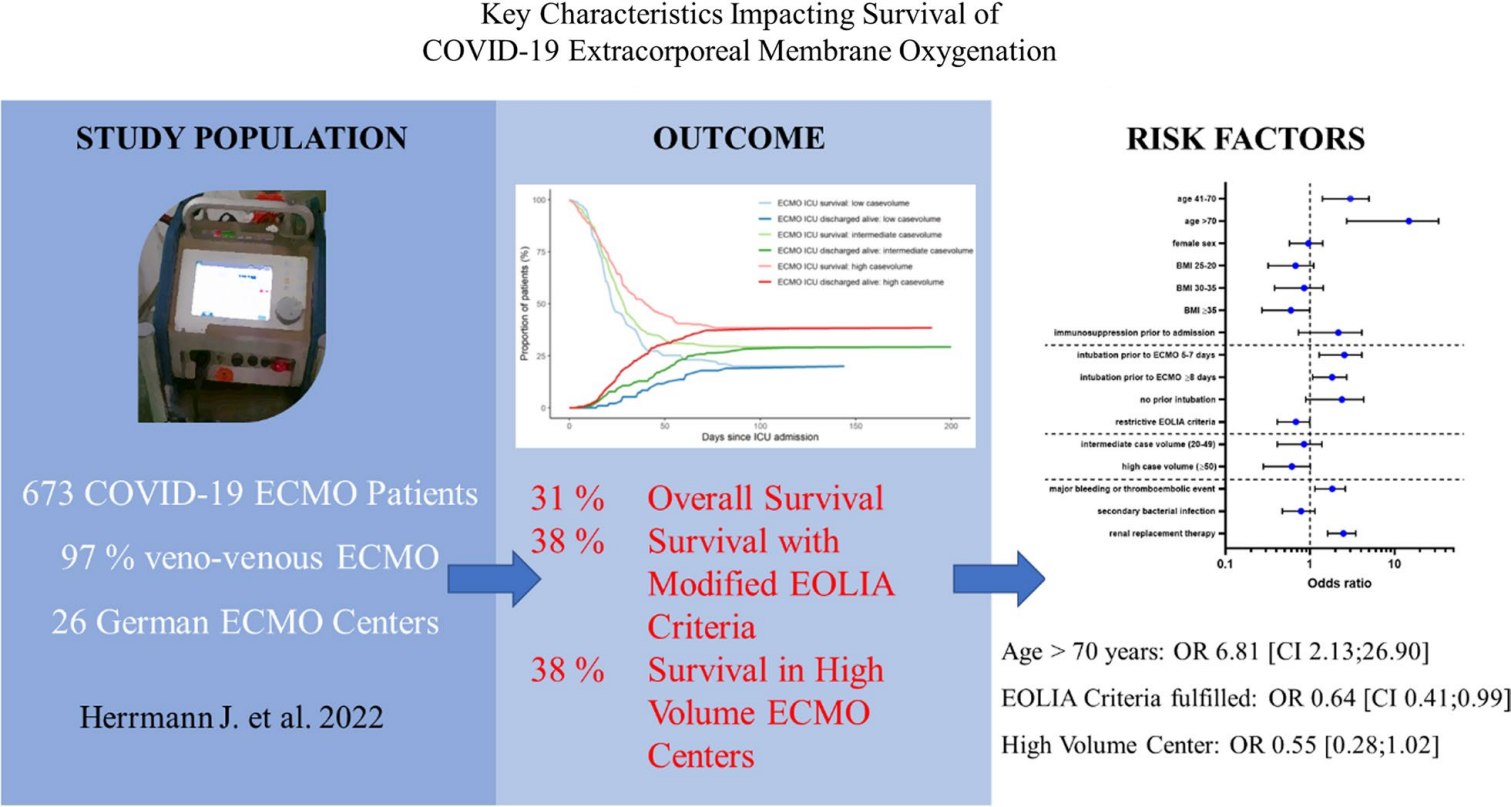

**Supplementary Information:**

The online version contains supplementary material available at 10.1186/s13054-022-04053-6.

## Introduction

The COVID-19 pandemic is challenging intensive care providers due to severe and prolonged cases of COVID-19 induced acute respiratory distress syndrome (ARDS). Compromised gas exchange may deteriorate despite maximum medical care, whereas veno-venous extracorporeal membrane oxygenation (VV ECMO) offers the chance to uphold oxygenation, carbon dioxide removal and rest the lungs. Although ECMO provides a rescue strategy and bridge to recovery, its use is resource intensive and can be associated with serious complications. In Germany, ECMO utilization had already increased manifold prior to the COVID-19 pandemic [1].

Need and indications of ECMO support are not universally defined but vary between centers and resource availability. In a pandemic ECMO use likely follows a U-shaped curve. A high number of patients are treated as COVID-19 numbers rise, decrease when hospital strain exceeds their capabilities and may rise again as strain eases [2]. In contrast to other countries, the German health care system was not overloaded during the COVID-19 pandemic [3]. In this context, patients with a lower, but reasonable probability of survival received ECMO support and numerous low to high volume ECMO centers treated COVID-19 ARDS patients. Recent health insurance data including more than 4000 VV ECMO patients surprisingly revealed a hospital survival rate of only 34%, thus further detailed structural and patient related analyses are urgently needed [4].

The continuous provision of organizational structures for successful ECMO therapy is challenging and during the pandemic less experienced centers have been faced with an increased number of ECMO patients, necessitating careful planning and training [5]. In this regard, effectiveness of low volume centers had already been questioned in non-COVID ECMO. In previous studies, admission to hospitals treating more than 30 [6] or more than 50 [7] ECMO patients per year was associated with a lower mortality in veno-arterial ECMO (VA ECMO). However, volume-outcome relationships have been less frequently defined in VV ECMO. An analysis of the Extracorporeal Life Support Organization Registry found no significant association between center volumes and patient survival in non-COVID respiratory assist [6]. Nonetheless, a position paper of renowned experts recommended that respiratory ECMO programs should treat at least 20 patients per year, including 12 respiratory cases [8]. Moreover, a recent study found that centers with longer experience with COVID-19 ECMO had a lower mortality rate relative to centers that started COVID-19 ECMO at a later timepoint [9].

We performed a multicenter study aiming to delineate the characteristics of ECMO therapy for COVID-19 induced ARDS, as well as to identify structural and patient-related factors independently associated with early survival of intensive care unit (ICU) treatment.

## Material and methods

### Study design and patient population

This is a retrospective observational study. Consecutive patients with SARS-CoV-2 infection confirmed with real-time reverse transcriptase polymerase chain reaction (RT-PCR) testing suffering from ARDS treated with ECMO at 26 ECMO centers across Germany between January 1st 2020 and March 22nd 2021 were included (Additional file 1: Figure S1). Hospitals in Germany utilizing COVID-19 ECMO support were invited to enter patient data into the register. The register continuously collects observational, multi-center data to recognize structural- and patient-related risk factors, complications, treatment effects and the outcome of COVID-19 ECMO patients.

### Indication of ECMO

Indications of ECMO support were at the discretion of the respective centers according to their in-house standards. Indications were classified as hypoxemia, hypercapnia, lung protective ventilation, right heart failure, left heart failure, cardiopulmonary resuscitation, or pulmonary embolism.

### Data collection and variable definition

Data were documented by the treating physicians within a standardized electronic case report form (RedCap®, Vanderbilt University).

Bleeding events were assessed according to definitions by Schulman et al. [10] and Kaatz et al. [11]. Thromboembolic events were included if diagnosed by standardized ultrasound examinations or CT scans.

### Outcomes and subgroups

The primary endpoint was survival at ICU discharge. Experience of the ECMO centers as a structural criterion was categorized according to the number of VV ECMO performed in 2019 as follows: low (< 20), intermediate (20–49) and high (≥ 50). In 6 centers this information was not available, thus, the center`s experience was estimated based in the number of COVID ECMOs in the observation period.

### Statistical analysis

Descriptive statistics are expressed as median (IQR) for continuous variables and as frequencies for categorical variables (including a category for missing data). Differences between groups were tested using the Mann–Whitney U test (continuous variables), *χ*^2^ test (categorical variables) or Fisher’s exact test (categorical variables with observed frequencies < 5), as appropriate. In a subgroup analysis, modified EOLIA trial inclusion criteria were applied to evaluate the impact of liberal vs. restrictive patient selection [12]. These subgroup criteria were defined as use of ECMO, age ≤ 70 years, mechanical ventilation for less than 8 days prior to ECMO, body mass index ≤ 45 kg/m^2^, absence of malignancies and no history of myocardial infarction, congestive heart failure, chronic pulmonary disease, and moderate to severe liver or kidney disease. We used logistic regression analyses to determine variables associated with mortality during stay at ICU and estimated odds ratios (ORs) with corresponding 95% confidence intervals (CIs). We selected variables a priori based on clinical background knowledge and assigned them to blocks reflecting the clinical course over time. We adjusted the models block-wise in four additional blocks: 1. demographics, risk factors and comorbidities (age, sex, BMI and immunosuppression within 6 months prior to admission); 2. severity of disease (intubation prior to ECMO and EOLIA criteria); 3. ECMO case volume, and 4. complications (major bleeding or thromboembolic events, secondary bacterial infection and renal replacement therapy). The quality of the models was assessed using the Akaike information criterion (AIC). Due to the high number of missing values within distinct variables, missingness was considered as a separate category in the primary analysis. We applied the following sensitivity analyses for the model: EOLIA criteria fulfilled or not, and complete case analysis. Data analysis was performed with R version 4.1.0. Statistical significance was determined at an *α* level of 0.05 (two-tailed).

### Ethics

The Ethics Committee of the Medical Faculty of the Julius-Maximilians-University of Wuerzburg approved the study protocol (131/20-me). Additional local ethics committee votes were obtained from each of the participating ECMO centers. According to German legislation, no informed consent for retrospective, anonymous data is required and informed consent was waived by the ethics committee. This study was registered in the German Clinical Trials Register (study ID: DRKS00022964, retrospectively registered, September 7th 2020, https://www.drks.de/drks_web/navigate.do?navigationId=trial.HTML&TRIAL_ID=DRKS00022964).

## Results

### Patient population

**Fig. 1:**
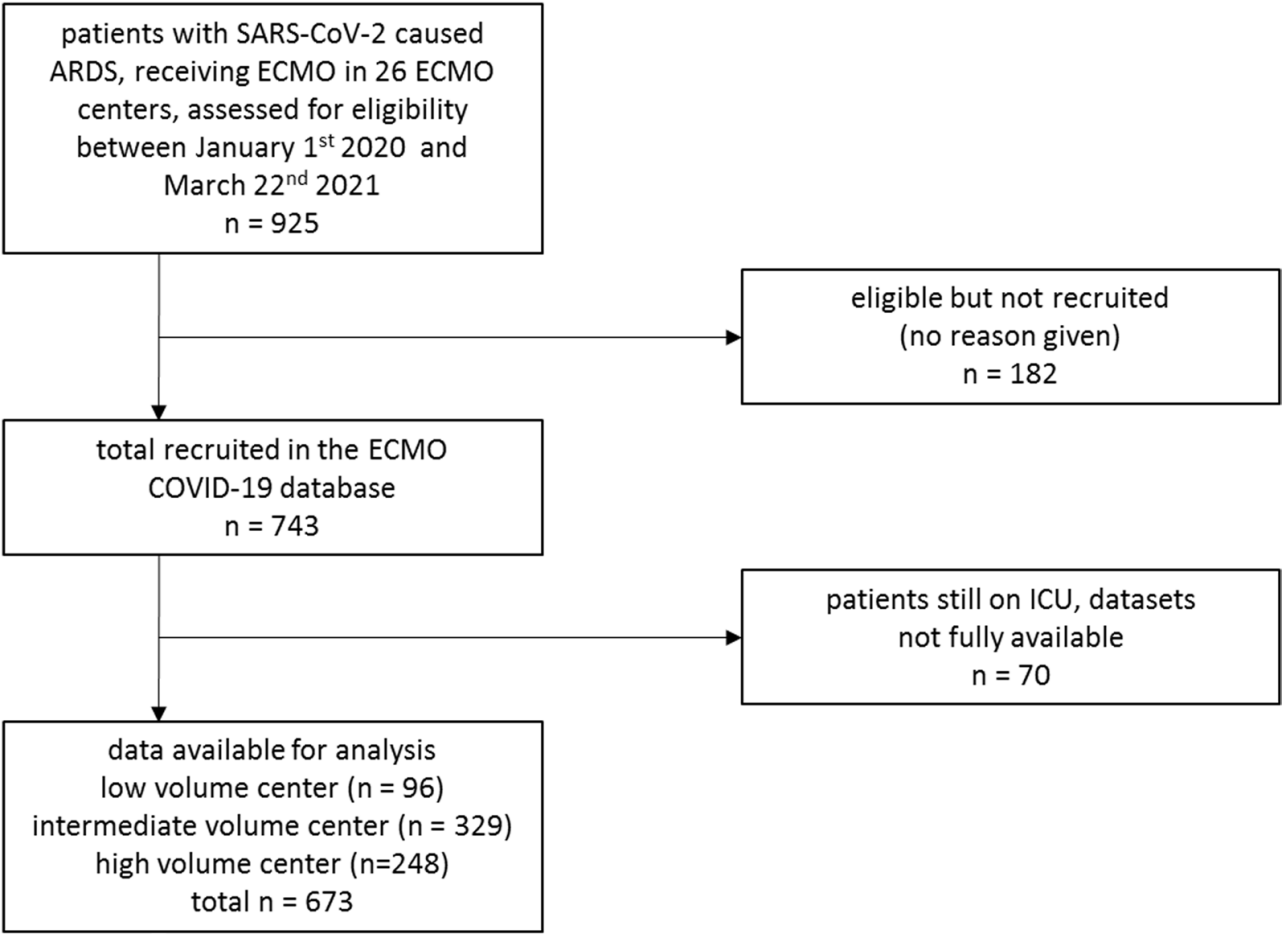
Flowchart patient recruitment

Of 925 patients treated between January 1st 2020 and March 22nd 2021, routine data from 743 patients were documented and 673 complete datasets were available at the first database closure on March 22, 2021 (Fig. 1). Intermediate volume centers treated 329 (49%), high volume centers 248 (37%), and low volume centers 96 (14%) patients.

Table 1(1) depicts demographics, risk factors and comorbidities. 535 (79%) were male, median BMI was 29 (IQR: 27–35). Most study patients were between 41 and 70 years (86%). The most frequent comorbidities were cardiovascular diseases (62%), diabetes mellitus (28%), and chronic pulmonary disease (15%). Immunosuppression was the only comorbidity significantly associated with mortality. 284 (42%) patients fulfilled modified EOLIA criteria, whereas 389 (58%) did not.

**Table 1:** Clinical characteristics of study cohort

*n*	Level	Overall—*n* (%)	Survivor—*n* (%)	Non-survivor—*n* (%)	*p*
		673 (100)	211 (31.4)	462 (68.6)	
*(1) Demographics, Risk factors, comorbidities*					
Date of hospital admission	04/2020–06/2020	186 (27.6)	75 (35.5)	111 (24.0)	0.0019
	07/2020–03/2021	487 (72.4)	136 (64.5)	351 (76.0)	
Age [years]	19–40	50 (7.4)	29 (13.7)	21 (4.6)	< 0.0001
	41–70	578 (85.9)	178 (84.4)	400 (86.6)	
	> 70	42 (6.2)	4 (1.9)	38 (8.2)	
	Missing	3 (0.4)	0 (0.00)	3 (0.7)	
Sex	m	535 (79.5)	161 (76.3)	374 (81,095)	0.3661
	w	131 (19.5)	48 (22.8)	83 (18.0)	
	Missing	7 (1.0)	2 (0.9)	5 (1.1)	
BMI [kg/m^2^]	< 25	81 (12.0)	21 (10.0)	60 (13.0)	0.0414
	25–30	263 (39.1)	83 (39.3)	180 (39.0)	
	30–35	147 (21.8)	43 (20.4)	104 (22.5)	
	≥ 35	157 (23.3)	61 (28.9)	96 (20.8)	
	Missing	25 (3.7)	3 (1.4)	22 (4.8)	
Cardiovascular disease	No	226 (33.6)	82 (38.9)	144 (31.2)	0.1323
	Yes	416 (61.8)	119 (56.4)	297 (64.3)	
	Missing	31 (4.6)	10 (4.7)	21 (4.6)	
Chronic pulmonary disease	No	564 (83.8)	179 (84.8)	385 (83.3)	0.8043
	Yes	103 (15.3)	31 (14.7)	72 (15.6)	
	Missing	6 (0.9)	1 (0.5)	5 (1.1)	
Diabetes mellitus	No	483 (71.8)	153 (72.5)	330 (71.4)	0.9401
	Yes	186 (27.6)	57 (27.0)	129 (27.9)	
	Missing	4 (0.6)	1 (0.5)	3 (0.7)	
Moderate to severe kidney disease	No	619 (92.0)	192 (91.0)	427 (92.4)	0.6161
	Yes	49 (7.3)	18 (8.5)	31 (6.7)	
	Missing	5 (0.7)	1 (0.5)	4 (0.9)	
Immunosuppression within 6 months prior to admission	No	523 (77.7)	158 (74.9)	365 (79.0)	0.0334
	Yes	39 (5.8)	8 (3.8)	31 (6.7)	
	Unknown	111 (16.5)	45 (21.3)	66 (14.3)	
*(2) Severity of disease, laboratory parameters (day 1)*					
EOLIA criteria	Met	284 (42.2)	108 (51.2)	176 (38.1)	0.0014
	Not met	389 (57.8)	103 (48.8)	286 (61.9)	
Indication of ECMO	Hypoxemia	419 (62.3)	126 (59.7)	293 (63.4)	0.5229
	Hypercapnia	141 (21.0)	43 (20.4)	98 (21.2)	
	Lung protective ventilation	48 (7.1)	16 (7.6)	32 (6.9)	
	Right heart failure	2 (0.3)	1 (0.5)	1 (0.2)	
	Left heart failure	4 (0.6)	1 (0.5)	3 (0.6)	
	Cardiopulmonary resuscitation	6 (0.9)	0 (0.0)	6 (1.3)	
	Pulmonary embolism	3 (0.4)	3 (1.4)	0 (0.0)	
	Other	9 (1.3)	5 (2.4)	4 (0.9)	
Intubation prior to ECMO [days]	< 5	275 (40.9)	102 (48.3)	173 (37.4)	0.0004
	5–7	102 (15.2)	20 (9.5)	82 (17.7)	
	≥ 8	179 (26.6)	44 (20.9)	135 (29.2)	
	No prior intubation	75 (11.1)	33 (15.6)	42 (9.1)	
	Missing	42 (6.2)	12 (5.7)	30 (6.5)	
Creatinine [mg/dl]	≤ 1.17	313 (46.5)	99 (46.9)	214 (46.3)	0.8757
	> 1.17	304 (45.2)	93 (44.1)	211 (45.7)	
	Missing	56 (8.3)	19 (9.00)	37 (8.0)	
Mode of ventilation	Spontaneous	18 (2.67)	9 (4.27)	9 (1.95)	0.3042
	Assisted	71 (10.55)	19 (9.00)	52 (11.26)	
	Controlled	544 (80.83)	170 (80.57)	374 (80.95)	
	Missing	40 (5.94)	13 (6.16)	27 (5.84)	
Lung compliance [ml/cm H_2_O]		25.5 [18.1, 34.2]	26.0 [17.6, 35.7]	25.4 [18.4, 33.9]	0.7168
*(3) ECMO and adjunct therapy*					
Mode of ECMO	VV	651 (96.7)	205 (97.2)	446 (96.5)	0.5104
	VA	12 (1.8)	2 (0.9)	10 (2.2)	
	VVA	10 (1.5)	4 (1.9)	6 (1.3)	
Cannula	Single lumen	544 (80.8)	153 (72.5)	391 (84.6)	0.0028
	Double lumen	52 (7.7)	22 (10.4)	30 (6.5)	
	Unknown	70 (10.4)	33 (15.6)	37 (8.0)	
	Missing	7 (1.0)	3 (1.4)	4 (0.9)	
Case volume ECMO center [*n*/year]	Low (< 20)	96 (14.3)	19 (9.0)	77 (16.7)	0.0024
	Intermediate (20–49)	329 (48.9)	97 (46.0)	232 (50.2)	
	High (≥ 50)	248 (36.9)	95 (45.0)	153 (33.1)	
Duration of ECMO support [h]		312.5 [144.0, 528.0]	336.0 [178.2, 560.8]	300.0 [120.0, 502.5]	0.0046
Prone positioning	No	240 (35.7)	64 (30.3)	176 (38.1)	0.1314
	Yes	333 (49.5)	111 (52.6)	222 (48.1)	
	Missing	100 (14.9)	36 (17.1)	64 (13.9)	
Therapeutic Anticoagulation	No	53 (7.9)	22 (10.4)	31 (6.7)	0.0968
	Yes	620 (92.1)	189 (89.6)	431 (93.3)	
*(4) Complications during ECMO*					
Major bleeding or thromboembolic event	No	231 (34.3)	97 (46.0)	134 (29.0)	< 0.0001
	Yes	442 (65.7)	114 (54.0)	328 (71.0)	
Secondary bacterial infection (respiratory tract or bloodstream)	No	239 (35.5)	81 (38.4)	158 (34.2)	0.2921
	Yes	434 (64.5)	130 (61.6)	304 (65.8)	
Renal replacement therapy	No	282 (41.9)	123 (58.3)	159 (34.4)	< 0.0001
	Yes	391 (58.1)	88 (41.7)	303 (65.6)	

Clinical characteristics of study cohort. Clinical characteristics in total population (*n* = 673) and survivor (*n* = 211) vs. non-survivor (*n* = 462). Parameters were slit up into blocks: (1) Demographics, Risk factors, Comorbidities; (2) Severity of Disease, Laboratory Parameters (day 1); (3) ECMO and adjunct therapy; (4) Complications during ECMO. Descriptive statistics are expressed as frequencies for categorical variables (including a category for missing data). Lung compliance is expressed as median (IQR) and data are missing for 34%, since the parameter was recorded in controlled ventilated patients only. Differences between groups were tested using the Mann–Whitney U test (continuous variables), *χ*^2^ test (categorical variables) or Fisher’s exact test (categorical variables with observed frequencies < 5), as appropriate

In low volume centers, less patients fulfilled modified EOLIA criteria and had higher SOFA scores at the time of ECMO initiation. During therapy, high volume centers reported significantly less bleeding/thromboembolic events, less secondary bacterial infections, and a lower need for renal replacement therapy (Table 2).

**Table 2:** Case volume-dependent characteristics in study cohort

	Low (< 20) *n*/(%)	Intermediate (20–49) *n*/(%)	High (≥ 50) *n*/(%)	*p*
Total	96 (14.3)	329 (48.9)	248 (36.9)	
Survivor	19 (19.8)	97 (29.5)	95 (38.3)	0.0024
EOLIA criteria fulfilled	27 (28.1)	146 (44.4)	111 (44.8)	0.0106
*SOFA (day 1)*				
0–9	32 (33.3)	31 (9.4)	64 (25.8)	0.0005
9–12	20 (20.8)	91 (27.7)	21 (8.5)	
12–15	15 (15.6)	71 (21.5)	4 (1.6)	
15–21	10 (10.4)	75 (22.8)	1 (0.4)	
Missing	19 (19.8)	61 (18.5)	158 (64.7)	
*Intubation prior to ECMO [days]*				
< 5	25 (26.0)	171 (52.0)	79 (32.9)	0.0005
5–7	21 (21.9)	51 (15.5)	30 (12.1)	
≥ 8	46 (47.9)	87 (26.4)	46 (18.5)	
No prior	1 (1.0)	9 (2.7)	65 (26.2)	
Missing	3 (3.1)	11 (3.3)	28 (11.3)	
Major bleeding or thromboembolic events	70 (72.9)	254 (77.2)	112 (45.2)	< 0.0001
Secondary bacterial infection (respiratory tract or bloodstream)	69 (71.9)	253 (76.9)	112 (45.2)	< 0.0001
Renal replacement therapy	64 (66.7)	208 (63.2)	119 (48.0)	0.0002

Survival, disease severity according to SOFA score, time between intubation and ECMO cannulation, incidence of complications (Major bleeding or thromboembolic events, secondary bacterial infection, renal replacement therapy) dependent on annual case volume. Results for SOFA score are given within ranges (0–9, 9–12, 12–15, 15–21). Descriptive statistics are expressed as frequencies for categorical variables (including a category for missing data). Differences between groups were tested using the Mann–Whitney U test (continuous variables), *χ*^2^ test (categorical variables) or Fisher’s exact test (categorical variables with observed frequencies < 5), as appropriate

### Pre-ECMO characteristics

Additional file 1: Table S1 shows parameters of gas exchange prior to ECMO initiation. Median PaO_2_/FiO_2_ ratio prior to ECMO initiation was 72 mmHg (IQR: 58–99), indicating severe ARDS according to the BERLIN definition [13]. There was no statistically significant difference in PaO_2_ (69 mmHg vs. 67 mmHg), SpO_2_ (92% vs. 91%) and pH (7.28 vs. 7.27) between survivors vs. non-survivors prior to ECMO (data not shown). On average, SARS-CoV-2 was diagnosed 2 days (IQR: 0–6) prior to ICU admission. Duration between hospital to ICU admission was 1 day (IQR: 0–3) (data not shown).

#### ECMO support

Table 1 and Additional file 1: Table S1 show severity of disease, parameters of mechanical ventilation, ECMO support and adjunct therapies. ECMO support started with a median of 5 days (IQR 1–9) after endotracheal intubation (data not shown). Mobile teams transported 493 patients (73%) from peripheral hospitals to designated ARDS/ECMO centers. Time between intubation and commencement of ECMO support was longer in low compared to high volume centers. Duration between intubation and start of ECMO support was significantly different between survivors (2 days, IQR 1–8) and non-survivors (5 days, IQR 2–9). Most patients were cannulated due to hypoxemia (62%) or hypercapnia (21%). In almost 97% of the cases, VV ECMO was the mode of choice. The most frequent cannulation side was “cervical and femoral” (60%) (data not shown).

During ECMO support prone positioning was used in 49%, neuromuscular blockers in 42%, therapeutic anticoagulation in 92% and glucocorticoids in 67% of the patients. 58% received renal replacement therapy.

Severe complications were frequent during ECMO therapy. Major bleeding or thromboembolic events occurred in 66% of the patients. Moreover, secondary bacterial respiratory tract or blood stream infections occurred in 64%. Major bleeding or thromboembolic events and renal replacement therapy were significantly associated with poor survival (Table 1). During therapy, high volume centers reported significantly less bleeding/thromboembolic events, less secondary bacterial infections, and a lower need for renal replacement therapy (Table 2).

### Outcome

**Fig. 2:**
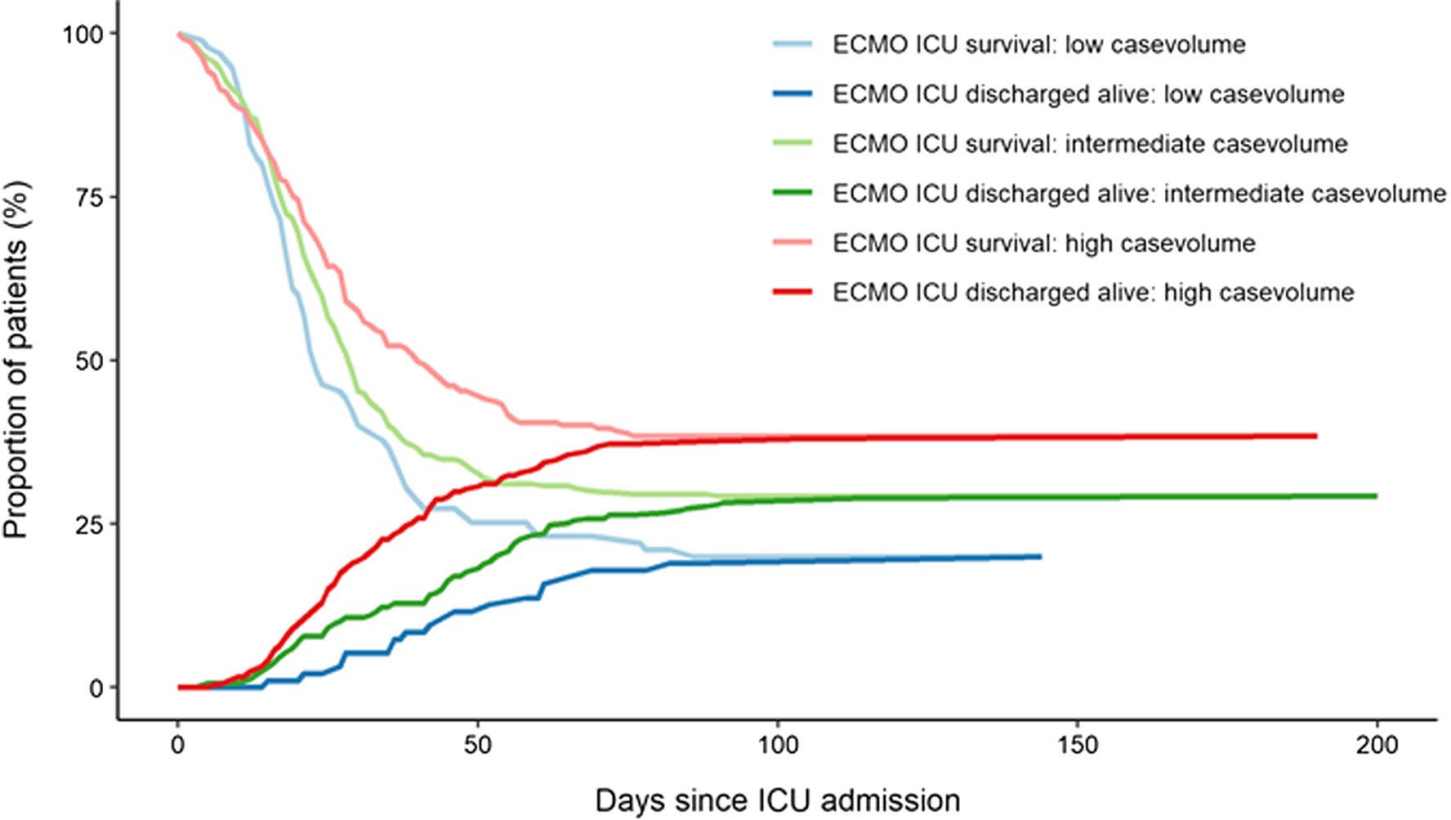
Volume-outcome relationship of COVID-19 ECMO. Case volume. Case volume vs. survival in low (*n* = 96, survival 20%), intermediate (*n* = 329, survival 30%) and high volume (*n* = 248, survival 38%) ECMO centers. Annual case volumes prior to the pandemic were defined as low (< 20/year), intermediate (20–49/year) and high (> 50/year). Lower lines (ECMO ICU discharged alive) depict the percentage of patents discharged alive from the ECMO providing ICU. ICU discharge destinations were mainly other ICUs (40%), rehabilitation facilities (33%), or general wards (23%) (data not shown)

Overall survival to ICU discharge was 31.4%. During the 1st wave of the COVID-19 pandemic (04/2020-06/2020) 40.3% survived to ICU discharge, while survival was significantly lower during the 2nd wave of the COVID-19 pandemic (07/2020-03/2021, 27.9%, *p* = 0.0019; Table 1(1), Additional file 1: Figure S2). ICU survival was higher, when ECMO therapy started within 5 days after endotracheal intubation (Table 1). Survival was significantly different between patients fulfilling modified EOLIA inclusion criteria (38.0%) and patients not fulfilling these criteria (26.5%) (*p* = 0.0014) (Table 1). Characteristics of the respective patient cohorts are found in Additional file 1: Table S2 and S3. Moreover, survival differed according to age group, ranging from 58.0% (19–40 years) to 9.5% (71–80 years) (Table 1). Survival also significantly differed according to case volume and was 20% for low volume centers, 30% for intermediate volume centers and 38% for high volume centers (*p* = 0.0024), respectively (Fig. 2).

### Factors affecting survival

**Fig. 3:**
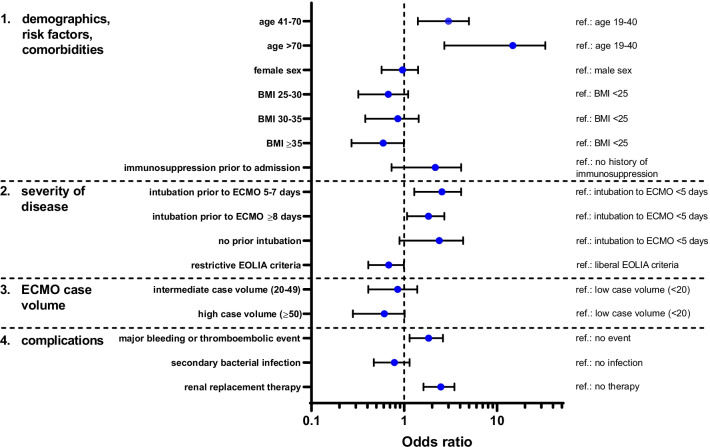
Risk factors for mortality. Adjusted odds ratios (OR) and 95% confidence intervals (CI) of risk factors for mortality according to the final model of block wise logistic regression (Table 3, Block 1–4). References for each variable are indicated (ref)

Results of the univariate logistic regression and block wise logistic regression, containing aspects of demographics, comorbidities, disease severity, ECMO therapy and complications are depicted in Table 3 and Fig. 3. Independent risk factors for non-survival included higher age with an OR of 2.48 (CI 1.32–4.17)) in patients aged 41–70 years and 6.81 (CI 2.13–26.90) in patients aged 71–80 compared to 19–40 years. Time periods of 5–7 days between intubation and ECMO initiation resulted in higher mortality (OR 2.39; CI 1.35–4.37) compared to < 5 days, whereas ≥ 8 days was not an independent risk factor (OR 1.30; CI 0.76–2.22). Patients fulfilling modified EOLIA criteria had an improved chance of survival with an OR of 0.64 (CI 0.41–0.99). Higher case volume of the ARDS/ECMO center in the preceding year also led to improved chances of survival with an OR of 0.55 (CI 0.28–1.02) for high compared to low volume centers. Interestingly, BMI ≥ 35 compared to BMI < 25 was also associated with higher chances of survival (OR 0.51; CI 0.26–0.97). Furthermore, major bleeding or thromboembolic events resulted in an OR of 1.70 (CI 1.12–2.57) for non-survival. Renal replacement therapy (OR 2.35; CI 1.60–3.46) was also an independent risk factor of mortality.

**Table 3:** Blockwise logistic regression

Blocks adjusted		Block 1		Block 1–2		Block 1–3		Block 1–4	
Variable	Units	OR	CI 95	OR	CI 95	OR	CI 95	OR	CI 95
*1. Demographics, risk factors, comorbidities*									
Age [years]	19–40	Ref		Ref		Ref		Ref	
	41–70	3.11	[1.71;5.78]	2.75	[1.49;5.15]	2.71	[1.46;5.10]	2.48	[1.32;4.71]
	> 70	12.31	[4.07;46.66]	8.70	[2.78;33.78]	8.28	[2.63;32.25]	6.81	[2.13;26.90]
	Missing	2,329,070.78	[0.00;NA]	2,976,862.44	[0.00;NA]	3,313,285.99	[0.00;NA]	1,813,873.83	[0.00;NA]
Sex	m	Ref		Ref		Ref		Ref	
	w	0.87	[0.57;1.34]	0.79	[0.51;1.24]	0.78	[0.50;1.23]	0.87	[0.55;1.38]
	Missing	0.89	[0.18;6.34]	1.06	[0.22;7.66]	0.97	[0.20;7.03]	1.12	[0.21;8.39]
BMI [kg/m^2^]	< 25	Ref		Ref		Ref		Ref	
	25–30	0.63	[0.35;1.13]	0.65	[0.35;1.17]	0.65	[0.35;1.18]	0.62	[0.33;1.13]
	30–35	0.70	[0.37;1.32]	0.71	[0.37;1.35]	0.73	[0.38;1.40]	0.74	[0.38;1.43]
	≥ 35	0.54	[0.29;0.99]	0.57	[0.30;1.07]	0.57	[0.30;1.06]	0.51	[0.26;0.97]
	Missing	2.49	[0.74;11.48]	3.16	[0.91;14.85]	3.13	[0.89;14.84]	3.25	[0.90;15.77]
Immunosuppression within 6 months prior to admission	No	Ref		Ref		Ref		Ref	
	Yes	1.82	[0.84;4.44]	1.69	[0.76;4.16]	1.75	[0.78;4.34]	1.44	[0.63;3.63]
	Unknown	0.61	[0.39;0.95]	0.73	[0.42;1.31]	0.76	[0.42;1.36]	0.77	[0.41;1.44]
*2. Severity of disease*									
Intubation prior to ECMO [days]	< 5			Ref		Ref		Ref	
	5–7			2.67	[1.54;4.81]	2.65	[1.52;4.81]	2.39	[1.35;4.37]
	≥ 8			1.31	[0.79;2.18]	1.26	[0.75;2.10]	1.30	[0.76;2.22]
	No prior intubation			0.88	[0.45;1.73]	1.12	[0.55;2.33]	1.90	[0.87;4.25]
	Missing			1.57	[0.76;3.43]	1.86	[0.88;4.13]	1.93	[0.89;4.42]
EOLIA criteria	Liberal			Ref		Ref		Ref	
	Restrictive			0.62	[0.40;0.95]	0.62	[0.40;0.95]	0.64	[0.41;0.99]
*3. ECMO Case volume*	Low					Ref		Ref	
	Medium					0.81	[0.44;1.44]	0.79	[0.42;1.43]
	High					0.53	[0.28;0.97]	0.55	[0.28;1.02]
*4. Complications*									
Major bleeding or thromboembolic event	No							Ref	
	Yes							1.70	[1.12;2.57]
Secondary bacterial infection (respiratory tract or bloodstream)	No							Ref	
	Yes							0.75	[0.48;1.16]
Renal replacement therapy	No							Ref	
	Yes							2.35	[1.60;3.46]
AIC		*815.24*		*804.94*		*803.01*		*780.89*	

Variables associated with mortality during ICU stay and odds ratios (ORs) with corresponding 95% confidence intervals (CIs) in blockwise logistic regression. Variables were selected a priori based on clinical background and assigned to blocks reflecting the clinical course over time. References for each variable are indicated (ref). Models were adjusted in four blocks: 1. Demographics, Risk factors and Comorbidities; 2. Severity of disease; 3. ECMO case volume, and 4. Complications. Step-wise integration of blocks into the model is depicted in the table from the left (block 1 only) to the right (block 1–4). The quality of the models was assessed using the Akaike information criterion (AIC). Statistical significance was determined at an *α* level of 0.05 (two-tailed)

## Discussion

In our study analyzing 673 patients treated in 26 German ECMO centers without resource constraints, 31.4% survived COVID-19 ECMO to ICU discharge. Patients younger than 40 years of age, without the need of renal replacement therapy, treated in a high volume ECMO center were most likely to survive COVID-19 ECMO. Moreover, patients without significant comorbidities, fulfilling modified EOLIA criteria had a significantly higher chance of survival. This emphasizes the importance of patient selection, identifying those that benefit the most.

VV ECMO use has rapidly increased during the COVID-19 pandemic with first studies indicating high chances of survival [14, 15]. Data from the Extracorporeal Life Support Organization (ELSO) Registry, Greater Paris, the US and Chile showed 90-day-survival rates ranging between 46 and 65% [14–18]. Accordingly, a recent meta-analysis reported a survival rate of 62.9% until hospital discharge [19]. In the current retrospective analysis, survival was lower but comparable with preliminary analyses of German health care insurance data, reporting in-hospital mortalities of 73% and 66% [4, 20]. However, survival depends on multiple factors including local resource allocation, patient inclusion criteria, timing of ECMO initiation, as well as experience of the centers. These factors varied between health care systems. Most importantly, none of the participating German ECMO centers experienced resource constraints or had to triage during the pandemic.

In our cohort, a high proportion of comorbid patients aged above 60 years were treated with ECMO. Although age per se is not an adequate cutoff parameter for any therapeutic intervention, our data indicate a very poor outcome in the elderly. Risk of non-survival progressively increased with age. Only 9.5% of COVID-19 patients older than 70 years survived ECMO therapy. Multiple studies have confirmed that increasing age is associated with a higher risk of death which has been explained by an increasing number of comorbidities [5, 14, 15, 17, 20]. However, only immunosuppression within 6 months prior to admission was associated with decreased survival. Therefore, increasing age can be considered as an independent risk factor and COVID-19 patients older than 70 years are significantly less likely to survive ECMO treatment.

To further delineate the impact of patient selection, we applied modified inclusion criteria of the EOLIA trial to our dataset by excluding patients older than 70 years and significant comorbidities. These patients accounted for a total of 42% of the study cohort, whereas survival significantly increased to 38.0%. Nevertheless, survival was still lower compared to a similar cohort from Greater Paris [15]. Hence, additional factors must explain this discrepancy. A larger proportion of patients not fulfilling the modified EOLIA criteria were treated at low volume centers (72%) compared to high volume centers (55%), suggesting that high volume centers used stricter ECMO inclusion criteria. High volume centers selected patients with lower scores of organ dysfunction/failure, started ECMO support earlier and had lower complication rates. Most importantly, chance of survival was doubled in high compared to low volume centers. It has previously been demonstrated that ECMO treatment in specialized high-volume ECMO centers benefits survival in non-COVID-19 ARDS [22, 23]. Similar, non ECMO critically ill patients also benefit from treatment in high volume centers [24]. Furthermore, early adopting hospitals starting COVID-19 ECMO prior to May 2020 have been shown to achieve better outcomes compared to new centers performing [9]. Most patients were treated during the 2nd wave of the pandemic when first virus variants were observed [21]. In situations requiring specific care and technical complex therapies such as ECMO, the outcome-volume relationship is pronounced, and organizational challenges may exaggerate during the COVID-19 pandemic. Hence, our data support the conclusion that patients should be treated in high-volume centers whenever possible.

In addition, commencement of ECMO therapy < 5 days after intubation is desirable. Mortality increased if ECMO started later than this time point. These results are in line with data from Greater Paris [15]. However, considering the results of the multivariate analysis, the effect of early ECMO initiation is interdependent with other contextual factors. In similar fashion, our results regarding a beneficial effect of class II obesity are limited. BMI > 35 was only compared to BMI < 25 and not against healthy weight and underweight patients. Nevertheless, our result is in line with current evidence suggesting improved 90-day survival in obese COVID-19 ECMO patients [25] or corroborating that obesity is not a risk factor for a worse outcome [26].

Considering complications of ECMO therapy, major bleeding or thromboembolic events were frequent and contributed to a poor outcome. ECMO requires systemic anticoagulation and bleeding dominates coagulation abnormalities [27]. Thromboembolic events did not significantly alter survival, although they have been associated with a higher risk of death in COVID-19 [28].

The use of renal replacement therapy was also associated with higher odds of non-survival. Contribution of ECMO to acute kidney injury is unknown [29], however, independent of ECMO, kidney failure is a marker of disease severity and associated with increased COVID-19 in-hospital mortality [30, 31]. Early detection of renal dysfunction in COVID-19 ARDS is crucial and the presence of renal replacement can be included as a risk factor of non-survival when evaluating the chances of bridging to recovery.

Strengths of our study include a large patient sample on a nationwide level recruited from low to high volume ECMO centers without resource constraints. Highly granular patient data collected during the entire course of ECMO support allowed a comprehensive analysis of risk factors. Sensitivity analysis allowed the evaluation of data quality and block wise regression permitted the identification of independent risk factors of non-survival (Additional file 1: Table S4). Limitations include the impossibility of external validation of the submitted patient data or structural criteria of the participating centers. Approximately 200 German hospitals performed COVID-19 ECMO [32], of which 26 entered data in the COVID-19 ECMO register, mainly due to the lack of staffing. Our dataset did not include changes of ECMO or cannulation modes during treatment. Moreover, our observation period was limited to intensive care in the ECMO providing ICU and did not include onset of COVID-19 related symptoms, time-to-event data, long-term follow-up, or cause of death.

## Conclusion

Careful selection of patients and high standards of care are necessary to maximize and justify ECMO support in COVID-19 ARDS. Survival of COVID-19 ECMO was underwhelming in elderly patients, in patients not fulfilling modified EOLIA inclusion criteria as well as those treated in low volume ECMO centers. These factors combined with a more liberal ECMO indication during the 2nd wave may explain the reasonably overall low survival rate. The observed volume-outcome relationship further suggests that ECMO allocation should prefer intermediate to high volume centers and patients should be transported to these centers whenever possible.

## Supplementary Information

**Additional file 1.** Supplemental data including participating centers, complete case analysis, delineation of the first and second wave of the pandemic, details on clinical course, as well as patient characteristics according to the use of modified EOLIA criteria.

## Data Availability

The data that support the findings of this study are available from the corresponding author, upon reasonable request.
