# Supplementary material for: Key characteristics impacting survival of COVID-19 extracorporeal membrane oxygenation
Source: Crit Care. 2022 Jun 28;26:190. doi: 10.1186/s13054-022-04053-6 (PMC9238175; doi:10.1186/s13054-022-04053-6)
Supplement: Supplementary file 1 — Additional file 1. Supplemental data including participating centers, complete case analysis, delineation of the first and second wave of the pandemic, details on clinical course, as well as patient characteristics according to the use of modified EOLIA criteria. [file 13054_2022_4053_MOESM1_ESM.docx]

**Additional file 1**

**Figure S1: Participating ECMO centers across Germany**


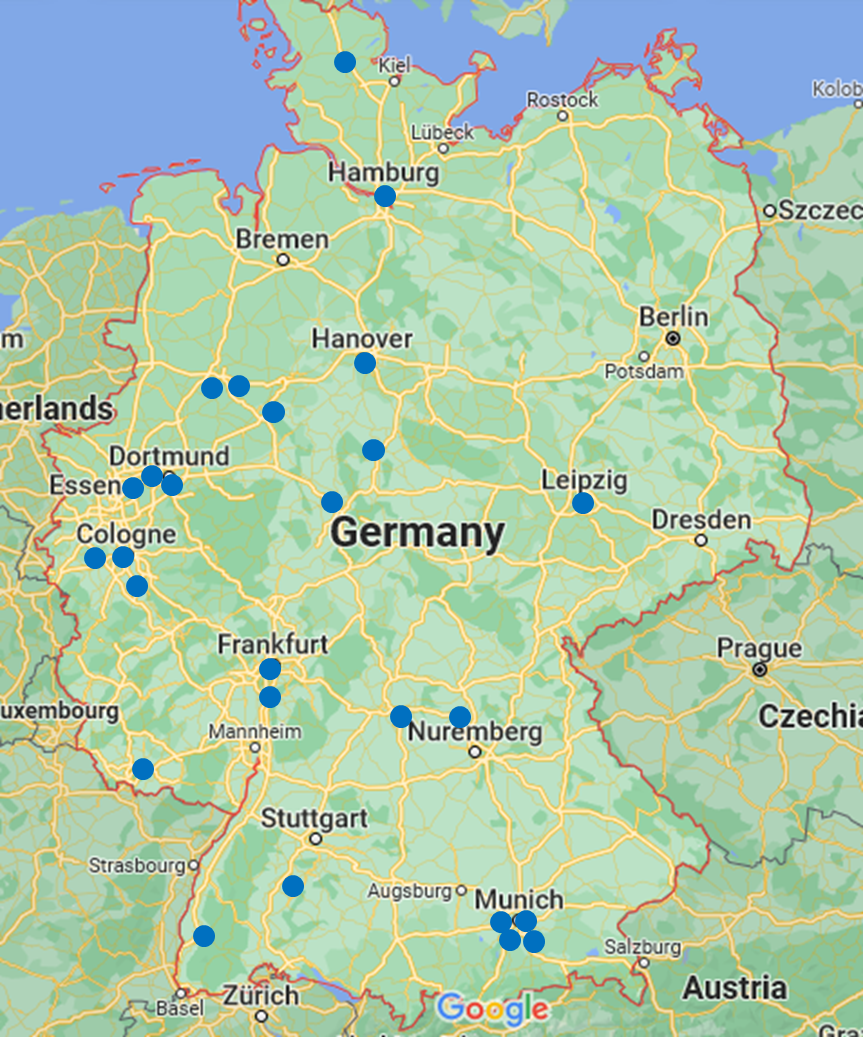


Figure S1: Participating ECMO centers across Germany

Locations of all 26 geographically diverse ECMO centers across Germany, which participated in the study.

**Figure S2: Survival and case numbers during first and second wave** **of the pandemic**

Figure S2**:** Survival and case numbers during the first and second wave of the pandemic.

ICU admission between January and June 2020 was defined as first wave and between July 2020 and March 2021 as the second wave.

**Table S1: ECMO characteristics during time course of therapy**

|  | **pre ECMO** | | | | **day 1** | | | | **day 3** | | | | **day 5** | | | |
| --- | --- | --- | --- | --- | --- | --- | --- | --- | --- | --- | --- | --- | --- | --- | --- | --- |
|  | overall | survivor | non-survivor | p | overall | survivor | non-survivor | p | overall | survivor | non-survivor | p | overall | survivor | non-survivor | p |
| p_a_O_2_/F_i_O_2_ [mmHg] | 72.1 (58.0-98.9) | 71.9 (56.8-104.0) | 72.3 (58.9-96.4) | 0.8847 | 135.6 (85.7-195.0) | 140.2 (83.8-197.1) | 133.2 (86.1-195.0) | 0.6478 | 125.5 (78.8-180.6) | 130.7 (80.4-197.1) | 122.8 (78.0-179.0) | 0.3102 | 110.3 (73.9-165.0) | 130.0 (76.1-804.4) | 104.0 (73.0-155.0) | 0.0063 |
| Sp_a_O_2_ [%] | 91.0 (85.0-95.0) | 92.0 (86.0-95.0) | 91.0 (85.0-95.0) | 0.1740 | 95.0 (92.8-97.0) | 95.1 (93.0-97.1) | 95.0 (92.6-97.0) | 0.1196 | 94.6 (92.0-96.4) | 95.0 (93.0-96.7) | 94.2 (91.7-96.2) | 0.0330 | 94.2 (91.9-96.0) | 95.0 (93.0-97.0) | 94.0 (91.2-96.0) | <0.0001 |
| Spontaneous breathing [n/%] | 18 (2.8) | 9 (4.6) | 9 (2.1) | 0.1645 | 10 (1.5) | 4 (1.9) | 6 (1.4) | 0.7794 | 11 (1.8) | 4 (1.9) | 7 (1.7) | 0.6544 | 13 (2.3) | 4 (2.1) | 9 (2.4) | 0.3016 |
| Assisted ventilation [n/%] | 71 (11.2) | 19 (9.6) | 52 (12.0) |  | 103 (15.7) | 31 (14.7) | 72 (16.2) |  | 126 (20.3) | 46 (22.3) | 80 (19.3) |  | 140 (24.6) | 55 (28.5) | 85 (22.6) |  |
| Controlled Mechanical ventilation [n/%] | 544 (85.9) | 170 (85.9) | 374 (86.0) |  | 543 (82.8) | 176 (83.4) | 367 (82.5) |  | 483 (77.9) | 156 (75.7) | 327 (79.0) |  | 416 (73.1) | 134 (69.4) | 282 (75.0) |  |
| F_i_O_2_ [n/%] | 100.0 (89.0-100.0) | 100.0 (90.0-100.0) | 100.0 (87.0-100.0) | 0.3697 | 60.0 (45.0-100.0) | 60.0 (43.0-100.0) | 60.0 (45.0-100.0) | 0.6903 | 60 (43.0-100.0) | 60.0 (40.0-100.0) | 65.0 (45.0-100.0) | 0.2270 | 70.0 (45.0-100.0) | 60.0 (40.0-100.0) | 70.0 (50.0-100.0) | 0.0631 |
| Lung Compliance [ml/cm H_2_O] | 25.5 (18.1-34.2) | 26.1 (17.5-35.8) | 25.4 (18.3-34.0) | 0.7171 | 23.5 (16.0-32.2) | 25.8 (17.5-33.8) | 22.7 (15.3-30.6) | 0.0095 | 21.2 (13.5-29.6) | 22.8 (15.5-30.0) | 20.4 (12.5-29.4) | 0.0537 | 20.0 (12.1-29.5) | 23.1 (15.7-33.2) | 17.4 (10.8-27.1) | <0.0001 |

|  | **day 7** | | | | **day 14** | | | | **last day of ICU stay** | | | |
| --- | --- | --- | --- | --- | --- | --- | --- | --- | --- | --- | --- | --- |
|  | overall | survi-vor | non-survi-vor | p | overall | survi-vor | non-survi-vor | p | overall | survi-vor | non-survi-vor | p |
| p_a_O_2_/F_i_O_2_ [mmHg] | 112.3 (72.1-173.3) | 121.8 (71.4-181.4) | 111.4 (72.5-157.8) | 0.2370 | 107.7 (74.5-167.5) | 125.5 (73.8-194.0) | 104.4 (74.6 147.5) | 0.0638 | 105.2 (69.5-190.1) | 192.3 (103.3-263.3) | 98.0 (68.4-177.5) | 0.0002 |
| Sp_a_O_2_ [%] | 94.2 (91.9-96.4) | 95.0 (93.0-97.0) | 93.8 (91.0-96.0) | <0.0001 | 94.6 (92.0-97.0) | 95.6 (93.5-97.2) | 94.0 (90.9-96.2) | <0.0001 | 94.9 (91.0-97.0) | 97.0 (95.0-98.3) | 93.2 (89.0-96.0) | <0.0001 |
| Spontaneous breathing [n/%] | 13 (2.5) | 5 (2.8) | 8 (2.3) | 0.0467 | 18 (4.7) | 12 (8.8) | 6 (2.4) | 0.0048 | 112 (18.3) | 96 (49.2) | 16 (3.8) | <0.0001 |
| Assisted ventilation [n/%] | 140 (26.7) | 60 (33.2) | 80 (23.3) |  | 101 (26.4) | 41 (30.2) | 60 (24.3) |  | 102 (16.6) | 39 (20.0) | 63 (15.1) |  |
| Controlled Mechanical ventilation [n/%] | 371 (70.8) | f116 (64.1) | 255 (74.3) |  | 264 (68.9) | 83 (61.0) | 181 (73.3) |  | 399 (65.1) | 60 (30.8) | 339 (81.1) |  |
| F_i_O_2_ [%] | 67.5 (50.0-100.0) | 60.0 (45.0-100.0) | 70.0 (50.0-100.0) | 0.6556 | 70.0 (46.0-100.0) | 60.0 (40.0-100.0) | 70.0 (50.0-100.0) | 0.2113 | 80.0 (45.0-100.0) | 40.0 (30.0-60.0) | 80.0 (50.0-100.0) | <0.0001 |
| Lung Compliance [ml/cm H_2_O] | 19.2 (11.4-31.6) | 19.4 (12.2-37.3) | 16.9 (10.0-30.0) | 0.0007 | 16.7 (9.3-25.5) | 19.4 (12.2-37.3) | 14.5 (8.4-23.1) | 0.0028 | 14.2 (7.5-25.0) | 32.1 (22.7-62.1) | 12.9 (7.0-21.9) | <0.0001 |

Table S1: ECMO characteristics during time course of therapy

Characteristics of ECMO therapy previous to cannulation (pre ECMO) and in course of treatment (day 1, day 3, day 5, day7, day 14, last day of ICU stay) in survivor (n=211) vs. non survivor (n=462). Units are indicated in squared brackets. Values in round brackets show median and IQR for continuous variables and frequencies for categorical variables. Descriptive statistics are expressed as median and IQR for continuous variables and as frequencies for categorical variables. Differences between groups were tested using the Mann-Whitney U test (continuous variables), χ2 test (categorical variables) or Fisher´s exact test (categorical variables with observed frequencies < 5), as appropriate.

**Table S2: Clinical characteristics of patients fulfilling modified EOLIA criteria**

|  | level | overall – n (%) | survivor – n (%) | non-survivor – n (%) | p |
| --- | --- | --- | --- | --- | --- |
| n |  | 284 (100.0) | 108 (38.0) | 176 (62.0) |  |
| **1. Demographics, Risk factors, Comorbidities** | | | | | |
| Date of hospital admission | 04/2020 -06/2020 | 63 (22.2) | 33 (30.6) | 30 (17.0) | 0.0078 |
|  | 07/2020 -03/2021 | 221 (77.8) | 75 (69.4) | 146 (83.0) |  |
| Age [years] | 19-40 | 30 (10.6) | 21 (19.4) | 9 (5.1) | 0.0002 |
|  | 41-70 | 251 (88.4) | 87 (80.6) | 164 (93.2) |  |
|  | >70 | 0 (0.0) | 0 (0.0) | 0 (0.0) |  |
|  | missing | 3 (1.1) | 0 (0.0) | 3 (1.7) |  |
| Sex | m | 227 (79.9) | 83 (76.9) | 144 (81.8) | 0.4911 |
|  | w | 53 (18.7) | 24 (22.2) | 29 (16.5) |  |
|  | missing | 4 (1.4) | 1 (0.9) | 3 (1.7) |  |
| BMI [kg/m^2^] | <25 | 32 (11.3) | 10 (9.3) | 22 (12.5) | 0.0485 |
|  | 25-30 | 114 (40.1) | 42 (38.9) | 72 (40.9) |  |
|  | 30-35 | 59 (20.8) | 24 (22.2) | 35 (19.9) |  |
|  | ≥35 | 65 (22.9) | 31 (28.7) | 34 (19.3) |  |
|  | missing | 14 (4.9) | 1 (0.9) | 13 (7.4) |  |
| Cardiovascular disease | no | 125 (44.0) | 55 (50.9) | 70 (39.8) | 0.1260 |
|  | yes | 142 (50.0) | 49 (45.4) | 93 (52.8) |  |
|  | missing | 17 (6.0) | 4 (3.7) | 13 (7.4) |  |
| Chronic pulmonary disease | no | 281 (98.9) | 107 (99.1) | 174 (98.9) |  |
|  | yes | 0 (0.0) | 0 (0.0) | 0 (0.0) |  |
|  | missing | 3 (1.1) | 1 (0.9) | 2 (1.1) |  |
| Diabetes mellitus | no | 213 (75.0) | 83 (76.9) | 130 (73.9) | 0.7968 |
|  | yes | 69 (24.3) | 24 (22.2) | 45 (25.6) |  |
|  | missing | 2 (0.7) | 1 (0.9) | 1 (0.6) |  |
| Moderate to severe kidney disease | no | 281 (98.9) | 107 (99.1) | 174 (98.9) |  |
|  | yes | 0 (0.0) | 0 (0.0) | 0 (0.0) |  |
|  | missing | 3 (1.1) | 1 (0.9) | 2 (1.1) |  |
| Immunosuppression within 6 months prior to admission | no | 229 (80.6) | 85 (78.7) | 144 (81.8) | 0.6892 |
|  | yes | 9 (3.2) | 3 (2.8) | 6 (3.4) |  |
|  | unknown | 46 (16.2) | 20 (18.5) | 26 (14.8) |  |
| **2. Severity of Disease, Laboratory Parameters (day 1)** | | | | | |
| Indication of ECMO | hypoxemia | 193 (68.0) | 73 (67.6) | 120 (68.2) | 0.7662 |
|  | hypercapnia | 48 (16.9) | 16 (14.8) | 32 (18.2) |  |
|  | other | 25 (8.8) | 11 (10.2) | 14 (8.0) |  |
|  | missing | 18 (6.3) | 8 (7.4) | 10 (5.7) |  |
| Intubation prior to ECMO [days] | <5 | 155 (54.6) | 64 (59.3) | 91 (51.7) | 0.0062 |
|  | 5-7 | 69 (24.3) | 15 (13.9) | 54 (30.7) |  |
|  | ≥8 | 0 (0.0) | 0 (0.0) | 0 (0.0) |  |
|  | no prior intubation | 34 (12.0) | 18 (16.7) | 16 (9.1) |  |
|  | missing | 26 (9.2) | 11 (10.2) | 15 (8.5) |  |
| Hemoglobin [g/dl] | <9 | 99 (34.9) | 32 (29.6) | 67 (38.1) | 0.2750 |
|  | ≥9 | 178 (62.7) | 74 (68.5) | 104 (59.1) |  |
|  | missing | 7 (2.5) | 2 (1.9) | 5 (2.8) |  |
| Creatinine [mg/dl] | ≤1.17 | 155 (54.6) | 56 (51.9) | 99 (56.3) | 0.6896 |
|  | >1.17 | 109 (38.4) | 43 (39.8) | 66 (37.5) |  |
|  | (Missing) | 20 (7.0) | 9 (8.3) | 11 (6.3) |  |
| **3. ECMO and adjunct therapy** | | | | | |
| Mode of ECMO | VV | 284 (100.0) | 108 (100.0) | 176 (100.0) |  |
|  | VA | 0 (0.0) | 0 (0.0) | 0 (0.0) |  |
|  | VVA | 0 (0.0) | 0 (0.0) | 0 (0.0) |  |
| Cannula | single lumen | 229 (80.6) | 80 (74.2) | 149 (84.7) | 0.0472 |
|  | double lumen | 24 (8.5) | 10 (9.3) | 14 (8.0) |  |
|  | unknown | 29 (10.2) | 16 (14.8) | 13 (7.4) |  |
|  | missing | 2 (0.7) | 2 (1.9) | 0 (0.0) |  |
| Case volume of ECMO center [n/year] | low (<20) | 27 (9.5) | 6 (5.6) | 21 (11.9) | 0.0406 |
|  | intermediate (20-49) | 146 (51.4) | 51 (47.2) | 95 (54.0) |  |
|  | high (≥50) | 111 (39.1) | 51 (47.2) | 60 (34.1) |  |
| Duration of ECMO support [h] |  | 288.0 [120.0, 528.0] | 316.0 [168.0, 588.2] | 268.5 [108.0, 499.0] | 0.0120 |
| Prone positioning | no | 86 (30.3) | 29 (26.9) | 57 (32.4) | 0.5931 |
|  | yes | 156 (54.9) | 63 (58.3) | 93 (52.8) |  |
|  | missing | 42 (14.8) | 16 (14.8) | 26 (14.8) |  |
| Therapeutic Anticoagulation | no | 23 (8.1) | 11 (10.2) | 12 (6.8) | 0.3126 |
|  | yes | 261 (91.9) | 97 (89.8) | 164 (93.2) |  |
| **4. Complications during ECMO** | | | | | |
| Major bleeding or thromboembolic event | no | 102 (35.9) | 48 (44.4) | 54 (30.7) | 0.0189 |
|  | yes | 182 (64.1) | 60 (55.6) | 122 (69.3) |  |
| Secondary bacterial infection (respiratory tract or bloodstream) | no | 105 (37.0) | 41 (38.0) | 64 (36.4) | 0.7864 |
|  | yes | 179 (63.0) | 67 (62.0) | 112 (63.6) |  |
| Renal replacement therapy | no | 129 (45.4) | 66 (61.1) | 63 (35.8) | <0.0001 |
|  | yes | 155 (54.6) | 42 (38.9) | 113 (64.2) |  |

Table S2: Clinical characteristics of patients fulfilling modified EOLIA criteria

Clinical characteristics in patients fulfilling modified EOLIA criteria (n=284) and survivor (n=108) vs. non-survivor (n=276). Parameters were slit up into blocks: 1. Demographics, Risk factors, Comorbidities; 2. Severity of Disease, Laboratory Parameters (day 1); 3 ECMO and adjunct therapy; 4. Complications during ECMO. Descriptive statistics are expressed as frequencies for categorical variables (including a category for missing data). Differences between groups were tested using the Mann-Whitney U test (continuous variables), χ2 test (categorical variables) or Fisher´s exact test (categorical variables with observed frequencies < 5), as appropriate.

**Table S3: Clinical characteristics of patients not fulfilling modified EOLIA criteria**

|  | level | overall – n (%) | survivor – n (%) | non-survivor – n (%) | p |
| --- | --- | --- | --- | --- | --- |
| n |  | 389 (100.0) | 103 (26.5) | 286 (73.5) |  |
| **1. Demographics, Risk factors, Comorbidities** | | | | | |
| Date of hospital admission | 04/2020 -06/2020 | 123 (31.6) | 42 (40.8) | 81 (28.3) | 0.0198 |
|  | 07/2020 -03/2021 | 266 (68.4) | 61 (59.2) | 205 (71.7) |  |
| Age [years] | 19-40 | 20 (5.1) | 8 (7.8) | 12 (4.2) | 0.0098 |
|  | 41-70 | 327 (84.1) | 91 (88.3) | 236 (82.5) |  |
|  | >70 | 42 (10.8) | 4 (3.9) | 38 (13.3) |  |
|  | missing | 0 (0.0) | 0 (0.0) | 0 (0.0) |  |
| Sex | m | 308 (79.2) | 78 (75.7) | 230 (80.4) | 0.4947 |
|  | w | 78 (20.1) | 24 (23.3) | 54 (18.9) |  |
|  | missing | 3 (0.8) | 1 (1.0) | 2 (0.7) |  |
| BMI [kg/m^2^] | <25 | 49 (12.6) | 11 (10.7) | 38 (13.3) | 0.4763 |
|  | 25-30 | 149 (38.3) | 41 (39.8) | 108 (37.8) |  |
|  | 30-35 | 88 (22.6) | 19 (18.4) | 69 (24.) |  |
|  | ≥35 | 92 (23.7) | 30 (29.1) | 62 (21.7) |  |
|  | missing | 11 (2.8) | 2 (1.9) | 9 (3.1) |  |
| Cardiovascular disease | no | 101 (26.0) | 27 (26.2) | 74 (25.9) | 0.3486 |
|  | yes | 274 (70.4) | 70 (68.0) | 204 (71.3) |  |
|  | missing | 14 (3.6) | 6 (5.8) | 8 (2.8) |  |
| Chronic pulmonary disease | no | 283 (72.8) | 72 (69.9) | 211 (73.8) | 0.4458 |
|  | yes | 103 (26.5) | 31 (30.1) | 72 (25.2) |  |
|  | missing | 3 (0.8) | 0 (0.0) | 3 (1.1) |  |
| Diabetes mellitus | no | 270 (69.4) | 70 (68.0) | 200 (69.9) | 0.8419 |
|  | yes | 117 (30.1) | 33 (32.0) | 84 (29.4) |  |
|  | missing | 2 (0.5) | 0 (0.0) | 2 (0.7) |  |
| Moderate to severe kidney disease | no | 338 (86.9) | 85 (82.5) | 253 (88.5) | 0.1792 |
|  | yes | 49 (12.6) | 18 (17.5) | 31 (10.8) |  |
|  | missing | 2 (0.5) | 0 (0.0) | 2 (0.7) |  |
| Immunosuppression within 6 months prior to admission | no | 294 (75.6) | 73 (70.9) | 221 (77.3) | 0.0353 |
|  | yes | 30 (7.7) | 5 (4.9) | 25 (8.7) |  |
|  | unknown | 65 (16.7) | 25 (24.3) | 40 (14.0) |  |
| **2. Severity of Disease, Laboratory Parameters (day 1)** | | | | | |
| Indication of ECMO | hypoxemia | 226 (58.1) | 53 (51.5) | 173 (60.5) | 0.4067 |
|  | hypercapnia | 93 (23.9) | 27 (26.2) | 66 (23.1) |  |
|  | other | 47 (12.1) | 15 (14.6) | 32 (11.2) |  |
|  | missing | 23 (5.9) | 8 (7.8) | 15 (5.2) |  |
| Intubation prior to ECMO [days] | <5 | 120 (30.9) | 38 (36.9) | 82 (28.7) | 0.0462 |
|  | 5-7 | 33 (8.5) | 5 (4.9) | 28 (9.8) |  |
|  | ≥8 | 179 (46.0) | 44 (42.7) | 135 (47.2) |  |
|  | no prior intubation | 41 (10.5) | 15 (14.6) | 26 (9.1) |  |
|  | missing | 16 (4.1) | 1 (1.0) | 15 (5.2) |  |
| Hemoglobin [g/dl] | <9 | 174 (44.7) | 44 (42.7) | 130 (45.5) | 0.0984 |
|  | ≥9 | 198 (50.9) | 58 (56.3) | 140 (49.0) |  |
|  | missing | 17 (4.4) | 1 (1.0) | 16 (5.6) |  |
| Creatinine [mg/dl] | ≤1.17 | 158 (40.6) | 43 (41.7) | 115 (40.2) | 0.9298 |
|  | >1.17 | 195 (50.1) | 50 (48.5) | 145 (50.7) |  |
|  | missing | 36 (9.3) | 10 (9.7) | 26 (9.1) |  |
| **3. ECMO and adjunct therapy** | | | | | |
| Mode of ECMO | VV | 367 (94.3) | 97 (94.2) | 270 (94.4) | 0.5492 |
|  | VA | 12 (3.1) | 2 (1.9) | 10 (3.5) |  |
|  | VVA | 10 (2.6) | 4 (3.9) | 6 (2.1) |  |
| Cannula | single lumen | 315 (81.0) | 73 (70.9) | 242 (84.6) | 0.0150 |
|  | double lumen | 28 (7.2) | 12 (11.7) | 16 (5.6) |  |
|  | unknown | 41 (10.5) | 17 (16.5) | 24 (8.4) |  |
|  | missing | 5 (1.3) | 1 (1.0) | 4 (1.4) |  |
| Case volume of ECMO center [n/year] | low (<20) | 69 (17.7) | 13 (12.6) | 56 (19.6) | 0.1068 |
|  | intermediate (20-49) | 183 (47.0) | 46 (44.7) | 137 (47.9) |  |
|  | high (≥50) | 137 (35.2) | 44 (42.7) | 93 (32.5) |  |
| Duration of ECMO support [h] |  | 341.0 [176.0, 520.0] | 352.5 [190.5, 552.0] | 340.0 [166.5, 507.0] | 0.2602 |
| Prone positioning | no | 154 (39.6) | 35 (34.0) | 119 (41.6) | 0.2166 |
|  | yes | 177 (45.5) | 48 (46.6) | 129 (45.1) |  |
|  | missing | 58 (14.9) | 20 (19.4) | 38 (13.3) |  |
| Therapeutic Anticoagulation | no | 30 (7.7) | 11 (10.7) | 19 (6.6) | 0.1880 |
|  | yes | 359 (92.3) | 92 (89.3) | 267 (93.7) |  |
| **4. Complications during ECMO** | | | | | |
| Major bleeding or thromboembolic event | no | 129 (33.2) | 49 (47.6) | 80 (28.0) | 0.0003 |
|  | yes | 260 (66.8) | 54 (52.4) | 206 (72.0) |  |
| Secondary bacterial infection (respiratory tract or bloodstream) | no | 134 (34.4) | 40 (38.8) | 94 (32.9) | 0.2744 |
|  | yes | 255 (65.6) | 63 (61.2) | 192 (67.1) |  |
| Renal replacement therapy | no | 153 (39.3) | 57 (55.3) | 96 (33.6) | 0.0001 |
|  | yes | 236 (60.7) | 46 (44.7) | 190 (66.4) |  |

Table S3: Clinical characteristics of patients not fulfilling modified EOLIA criteria

Clinical characteristics in patients not fulfilling modified EOLIA criteria (n=389) and survivor (n=103) vs. non-survivor (n=286). Parameters were slit up into blocks: 1. Demographics, Risk factors, Comorbidities; 2. Severity of Disease, Laboratory Parameters (day 1); 3 ECMO and adjunct therapy; 4. Complications during ECMO. Differences between groups were tested using the Mann-Whitney U test (continuous variables), χ2 test (categorical variables) or Fisher´s exact test (categorical variables with observed frequencies < 5), as appropriate.

# Table S4: Blockwise logistic regression – Complete case analysis (n=497)

| **Blocks adjusted** | | | **Block 1** | | **Block 1-2** | | **Block 1-3** | | **Block 1-4** | |
| --- | --- | --- | --- | --- | --- | --- | --- | --- | --- | --- |
|  | **variable** | **units** | **OR** | **CI 95** | **OR** | **CI 95** | **OR** | **CI 95** | **OR** | **CI 95** |
| **1. Demographics, risk factors, comorbidities** | **Age [years]** | 19-40 | ref |  | ref |  | ref |  | ref |  |
|  |  | 41-70 | 3.23 | [1.61; 6.62] | 2.98 | [1.46; 6.22] | 2.95 | [1.44; 6.18] | 2.65 | [1.25; 5.73] |
|  |  | >70 | 19.17 | [4.73; 131.06] | 16.83 | [4.07; 116.38] | 15.86 | [3.81; 110.05] | 10.96 | [2.55; 77.37] |
|  | **Sex** | m | ref |  | ref |  | ref |  | ref |  |
|  |  | w | 0.89 | [0.54; 1.49] | 0.83 | [0.50; 1.41] | 0.81 | [0.48; 1.37] | 0.96 | [0.56; 1.68] |
|  | **BMI [kg/m^2^]** | <25 | ref |  | ref |  | ref |  | ref |  |
|  |  | 25-30 | 1.08 | [0.56; 2.04] | 1.09 | [0.56; 2.10] | 1.07 | [0.54; 2.06] | 0.99 | [0.49; 1.95] |
|  |  | 30-35 | 1.03 | [0.51; 2.05] | 1.05 | [0.51; 2.14] | 1.05 | [0.51; 2.14] | 1.06 | [0.50; 2.22] |
|  |  | ≥35 | 0.87 | [0.43; 1.71] | 0.95 | [0.47; 1.91] | 0.92 | [0.45; 1.85] | 0.75 | [0.35; 1.56] |
|  | **Immunosuppression within 6 months prior to admission** | 0 | ref |  | ref |  | ref |  | ref |  |
|  |  | 1 | 1.77 | [0.77; 4.57] | 1.94 | [0.84; 5.08] | 2.06 | [0.88; 5.46] | 1.52 | [0.64; 4.08] |
| **2. Severity of disease** | **Intubation prior to ECMO [days]** | <5 |  |  | ref |  | ref |  | ref |  |
|  |  | 5-7 |  |  | 2.78 | [1.55; 5.21] | 2.79 | [1.55; 5.26] | 2.49 | [1.35; 4.76] |
|  |  | ≥8 |  |  | 1.83 | [1.16; 2.95] | 1.80 | [1.12; 2.93] | 1.89 | [1.14; 3.17] |
|  |  | no prior intubation |  |  | 0.91 | [0.31; 2.89] | 0.94 | [0.32; 2.97] | 1.07 | [0.35; 3.49] |
| **3. Case Volume of ECMO Center** | **[n/year]** | low (<20) |  |  |  |  | ref |  | ref |  |
|  |  | medium (20-49) |  |  |  |  | 0.92 | [0.48; 1.69] | 0.92 | [0.47; 1.75] |
|  |  | high (≥50) |  |  |  |  | 0.61 | [0.31; 1.16] | 0.58 | [0.29; 1.14] |
| **4. Complications** | **Major bleeding or thrombo-embolic event** | no |  |  |  |  |  |  | ref |  |
|  |  | yes |  |  |  |  |  |  | 2.00 | [1.27;3.16] |
|  | **Secondary bacterial infection (respiratory tract or bloodstream)** | no |  |  |  |  |  |  | ref |  |
|  |  | yes |  |  |  |  |  |  | 0.62 | [0.37;1.01] |
|  | **Kidney replacement therapy** | no |  |  |  |  |  |  | ref |  |
|  |  | yes |  |  |  |  |  |  | 2.90 | [1.88;4.50] |

Table S4: Blockwise logistic regression – Complete case analysis (n=497)

Variables associated with mortality during stay at ICU and odds ratios (ORs) with corresponding 95 % confidence intervals (CIs) in blockwise logistic regression. A complete case analysis with n=497 patients was performed as sensitivity analysis. Variables were selected a priori based on clinical background knowledge and assigned to blocks reflecting the clinical course over time. References for each variable are indicated (ref). Models were adjusted in four blocks: 1. Demographics, Risk factors and Comorbidities; 2. Severity of disease; 3. ECMO case volume, and 4. Complications. Step-wise integration of blocks into the model is depicted in the table from the left (block 1 only) to the right (block 1-4).
